# Empathy from dissimilarity: Multivariate pattern analysis of neural activity during observation of somatosensory experience

**DOI:** 10.1162/imag_a_00110

**Published:** 2024-03-19

**Authors:** Roshni Lulla, Leonardo Christov-Moore, Anthony Vaccaro, Nicco Reggente, Marco Iacoboni, Jonas T. Kaplan

**Affiliations:** Brain and Creativity Institute, Department of Psychology, University of Southern California, Los Angeles, CA, United States; Institute for Advanced Consciousness Studies, Santa Monica, Los Angeles, CA, United States; Ahmanson-Lovelace Brain Mapping Center, Department of Psychiatry and Biobehavioral Sciences, Semel Institute for Neuroscience and Human Behavior, Brain Research Institute, David Geffen School of Medicine at UCLA, Los Angeles, CA, United States

**Keywords:** empathy, affective experience, somatosensory resonance, representational dissimilarity, multivariate analysis

## Abstract

Empathy seems to rely on our ability to faithfully simulate multiple aspects of others’ inferred experiences, often using brain structures we would use during a similar experience. Much neuroimaging work in this vein has related empathic tendencies to univariate correlates of simulation strength or salience. However, novel evidence suggests that empathy may rely on the multivariate distinctiveness of these simulations. Someone whose representations of painful and non-painful stimulation are more distinct from each other may more accurately simulate that experience upon seeing somebody else experience it. We sought to predict empathic tendencies from the dissimilarity between neural activity patterns evoked by observing other people experience pain and touch and compared those findings to traditional univariate analyses. In support of a simulationist perspective, diverse observed somatosensory experiences were best classified by activation patterns in contralateral somatosensory and insular cortices, the same areas that would be active were the subject experiencing the stimuli themselves. In support of our specific hypothesis, the degree of dissimilarity between patterns for pain and touch in distinct areas was each associated with different aspects of trait empathy. Furthermore, the pattern dissimilarity analysis proved more informative regarding individual differences than analogous univariate analyses. These results suggest that multiple facets of empathy are associated with an ability to robustly distinguish between the simulated states of others at corresponding levels of the processing hierarchy, observable via the distinguishability of neural patterns arising with those states. Activation pattern dissimilarity may be a useful tool for parsing the neuroimaging correlates of complex cognitive functions like empathy.

## 1 Introduction

Empathy relies on affective, interoceptive, nociceptive, somatomotor, and cognitive processes that allow us to feel and infer, respectively, what another individual is experiencing (Christov-Moore & Iacoboni, 2016;Decety & Meltzoff, 2011;Hartmann et al., 2022;Keysers et al., 2010;Lamm et al., 2016;Rütgen et al., 2021). Converging evidence suggests that these processes rely on (and may in turn be biased) by our own feeling, experiencing and thinking machinery. Impaired somatic and affective experience as is seen in psychopathy and autism may limit empathic abilities (Blair et al., 2002;Marcoux et al., 2013;Sun et al., 2023). Healthy participants administered an analgesic have been found to be impaired in their ability to recognize and respond to others’ pain (Mischkowski et al., 2019;Rütgen et al., 2015). This effect was pronounced in a variety of studies combining electroencephalography as well as heart rate variability (De Pascalis & Vecchio 2022;Vecchio & De Pascalis 2021), which found that placebo analgesia reduced both self-pain and other pain. A parallel study showed that placebo analgesia reduced costly prosocial helping to reduce another’s pain. Contrasting studies show how comparing a placebo analgesic to control shows no differences in empathy, questioning the basis of somatosensory sharing (Hartmann et al., 2021). This identifies a clear need to further probe the mechanistic role of somatic experience in affective empathy.

Contemporary theories of brain organization propose a hierarchical representation of the world (Baldassano et al., 2017;Hilgetag & Goulag, 2020), where interconnected systems progressively encode more abstract and intricate aspects of our surroundings, spanning from basic sensory elements like edges and shapes to conceptual models of others, oneself, and the world. This ascending complexity parallels increased informational convergence (Meyer & Damasio, 2009), possibly mapped onto distinct brain regions. Optimal empathy may rely, in part, on the*granularity*of these internal representations, shedding light on the underlying processes of abstraction and convergence. It is also crucial to consider the involvement of different neural networks and whether the level of detail in neural representations within these networks can reliably distinguish between cognitive states. In the case of empathy, applying this logic to activation patterns evoked by somatic and affective stimuli may shed light on the mechanisms behind empathy’s instantiation in the brain.

Recent studies using MVPA find that distinct aspects of affect and cognition best correspond to specific*patterns*(rather than cluster-wise intensities) of neural activity (Celeghin et al., 2017;Nummenmaa & Saarimäki, 2019;Scarantino, 2012) in systems like medial prefrontal cortex (mPFC), inferior frontal gyrus (IFG), posterior medial cortex (PMC), insula, and amygdalae (Kim et al., 2015;Peelen et al., 2010;Saarimaki et al., 2016,^2018^;Sachs et al., 2018). Convergent representations of emotion have been found in mPFC and superior temporal sulcus, which appear to accurately classify emotions presented by either facial or bodily expression (Peelen et al., 2010). Activation patterns in somatosensory cortices have also been shown to robustly distinguish between externally expressed emotions (Cao et al., 2018;Kaplan & Meyer, 2012;Kryklywy et al., 2018;Sachs et al., 2018;Vaessen et al., 2019). These multivariate methods may provide insights above and beyond what is found with traditional univariate studies.

Our empathic understanding of others may arise from simulations of what it is like to be them. If, as many theories suggest, we represent the world via hierarchically-organized representations of features, then deficits (or high ability) in a given component of these representations may be reflected in our ability to simulate that type and level of representation in others. If we take this simulationist hypothesis seriously, it follows that people more prone to and able to engage empathy may have richer, more fine-grained internal models of others’ states generally, and, specifically, that the loci of that grain may inform the structure of their empathic abilities. In line with this inference, recent work has found that perspective taking is predicted by representational dissimilarity during affective experience in VMPFC (Vaccaro et al., 2022). We sought to apply this approach to study somatosensory simulations of others’ states.

Somatosensation, experienced and vicarious, relies on complex interactions between at least two systems, thought to subserve the affective and sensory/visceral components of pain and touch: an affective system, encompassing the anterior cingulate, paracingulate cortices, orbitofrontal, right dorsolateral and medial prefrontal cortices; and a somatosensory/visceral system, encompassing the somatomotor cortex and insula. This is supported both by studies dissecting the neural correlates of experienced somatosensation, and those dissecting the distinct neural correlates of properties of*observed*or*inferred*somatosensory experience (reviewed inLamm et al., 2011).

In the current study, we leveraged multivariate voxel pattern analysis (MVPA) to examine whether the dissimilarity of evoked responses to others’ somatosensory states are implicated in different facets of empathy, and study what the loci of dissimilarity can say about their underpinnings. 70 participants (36 female) filled out a four-scale measure of empathy (the Interpersonal Reactivity Index), and, within the MRI scanner, observed a human hand being pierced with a needle (Pain condition), poked lightly with a q-tip (Touch) or in isolation (Control), in counterbalanced order. In the first analysis, we aimed to determine which regions’ activation was most informative for classifying between three observed somatosensory stimulations (Pain, Touch, Control).

Following our general simulationist hypothesis (participants’ own areas for discriminating somatosensory stimulation to the observed area should be most informative), we anticipated that the contralateral (to the observed right hand) somatosensory and insular cortices would have the highest classification accuracy. We then conducted a dissimilarity analysis within a searchlight brain mapping procedure (Kriegeskorte, 2006) and regressed each participant’s empathy subscale scores against these dissimilarity maps, thus highlighting where this measure supported each facet of empathic function. We hypothesized that individual differences in the four empathy subcomponents of the IRI (empathic concern, personal distress, perspective-taking ability, and fantasizing) would be reflected in distinct loci of dissimilarity.

## 2 Materials and Methods

### 2.1 Participants

Participants were 70 ethnically diverse adults aged 18-35 (36 female) recruited from the local community through fliers. All recruitment and experimental procedures were performed under approval of UCLA’s institutional review board, in accordance with the ethical standards of the institutional and/or national research committee and with the 1964 Helsinki declaration and its later amendments or comparable ethical standards. Informed consent was obtained from all individual participants included in the study.

Eligibility criteria for participants included: right-handed, no prior or concurrent diagnosis of any neurological, psychiatric, or developmental disorders, and no history of drug or alcohol abuse. These were all assessed during preliminary screening interviews conducted by phone at the time of recruitment.

Due to the novel analysis performed, we were unable to perform a formal power analysis since the expected effect sizes could not be known. We aimed for a comparably large sample size balancing feasibility, cost, and participant availability. Our sample size is comparable to previous studies that conducted similar whole brain multivariate analyses, namely representational similarity analysis (RSA). A 2015 study that used RSA to understand the representation of social values and valence with emotional images had 48 participants, and a 2014 RSA study that similarly investigated affect had a sample size of only 16 (Chavez & Heatherton, 2015;Chikazoe et al., 2014). More recent studies that investigated social cognition such as mentalizing and creative thought had sample sizes of 61 and 35, respectively (Golec-Staśkiewicz et al., 2022;Matheson et al., 2023). However, we note that one should apply caution when comparing sample sizes across studies with different aims and possible effect sizes. It is important to acknowledge that by basing sample sizes off historical precedent rather than performing a formal power analysis may dampen and place an upper limit on the average power in the field of affective neuroscience. This can lead to further replication issues due to the “winner’s curse,” in which findings are artificially inflated because of lower-than-calculated power (Button et al., 2013).

#### 2.1.1 Stimuli

The stimuli were 27 full-color videos previously used byBufalari et al. (2007), and used with permission by their research group, depicting a human hand being pierced by a hypodermic syringe (Pain condition) and touched by a wooden q-tip (Touch condition) in varying locations, as well as a static hand without stimulation (Control condition). The experiment consisted of 12 trial blocks lasting 22 s each, plus 8 alternating rest blocks that lasted either 5 s or 10 s. Each trial block consisted of 4 videos of a single condition (Pain, Touch, Control), 5 s in duration each, with an interstimulus interval of 400 ms. Participants were simply instructed to watch the video clips and did not provide any responses within the scanner. They were assured that the hand in the video clip was a human hand and not a model, but they were not instructed to empathize with the hand’s owner nor were there any audiovisual cues to indicate pain in the hand’s owner. All stimuli shown to participants displayed a right hand.

For the task, two different block orders were used, and controlled to ensure an approximately equal proportion of male and female participants were exposed to each block order. The order of the fMRI and behavioral assessment was counterbalanced across participants. All tasks were coded within Presentation (Neurobehavioral Systems, Inc.).

#### 2.1.2 Behavioral assessment

After completing the fMRI portion of the experiment, participants filled out the following questionnaire in a closed room, unobserved.

*Interpersonal Reactivity Index (IRI)*: The IRI (Davis, 1980) is a widely used (Avenanti et al., 2009;Pfeifer et al., 2008) and validated (Litvack-Miller et al., 1997) questionnaire designed to measure both ‘‘cognitive’’ and ‘‘emotional’’ components of empathy. It consists of 24 statements that the participant rates on a 5-point scale ranging from 0 (Does not describe me very well) to 5 (Describes me very well). The statements are calculated to test four theorized subdimensions of empathy:

*Fantasizing Scale (FS)*: the tendency to take the perspective of fictional characters.

*Empathic Concern (EC)*: sympathetic reactions to the distress of others.

*Perspective Taking (PT)*: the tendency to take others’ perspective

*Personal Distress (PD)*: aversive reactions to the distress of others

Scores were summed for each sub-dimension (measured by 6 items) to make 4 scores per participant. Cronbach’s alpha, a measure of reliability, was assessed for the IRI using SPSS (FS = 0.756, EC = 0.773, PT = 0.807, PD = 0.821). The order of the questionnaire and the scanning session was counterbalanced across participants.

#### 2.1.3 fMRI data acquisition

fMRI data were acquired on a Siemens Trio 3 Tesla system housed in the Ahmanson-Lovelace Brain Mapping Center at UCLA. Functional images were collected over 36 axial slices covering the whole cerebral volume using an echo planar T2∗- weighted gradient echo sequence (TR = 2500 ms; TE = 25 ms; flip angle = 90∗; matrix size = 64 × 64; FOV 20 cm; in-plane resolution = 3 mm × 3 mm; slice thickness = 3 mm/1 mm gap). Additionally, a high-resolution T1-weighted volume was acquired in each participant (TR = 2300 ms; TE = 25 ms; TI = 100 ms; flip angle = 8∗; matrix size = 192 × 192; FOV = 256 cm; 160 slices), with approximately 1 mm isometric voxels (1.3 mm × 1.3 × 1.0 mm).

### 2.2 Preprocessing

Analyses were performed in FEAT (FMRI Expert Analysis Tool), part of FSL (FMRIB’s Software Library,http://www.fmrib.ox.ac.uk/fsl). After motion correction using MCFLIRT, images were temporally high-pass filtered with a cutoff period of 70 s for SE, (approximately equal to one rest-task-rest-task period) and smoothed using a 6 mm Gaussian FWHM algorithm in three dimensions. Each participant’s functional data was coregistered to standard space (MNI 152 template) via registration of an averaged functional image to the high-resolution T1-weighted volume using a six degree-of-freedom linear registration and of the high-resolution T1-weighted volume to the MNI 152 template via nonlinear registration, implemented in FNIRT. In order to remove non-neuronal sources of coherent oscillation in the relevant frequency band (0.01-0.1 Hz), preprocessed data were subjected to probabilistic independent component analysis as implemented in MELODIC (Multivariate Exploratory Linear Decomposition into Independent Components) Version 3.10, part of FSL (FMRIB’s Software Library). Noise components corresponding to head motion, scanner noise, and cardiac/respiratory signals were identified by observing their localization, time series, and spectral properties (as perKelly et al., 2010) and removed using FSL’s regfilt command.

#### 2.2.1 fMRI analysis

The BOLD response was modeled using an explanatory variable (EV) consisting of a boxcar function describing the onset and duration of each relevant experimental condition (task conditions, rest, instruction screen) convolved with a double gamma HRF to produce an expected BOLD response. The temporal derivative of each task EV was also included. Functional data were then fitted to the model using FSL’s implementation of the general linear model. This resulted in three normalized z statistic maps corresponding to each participant’s “activation” patterns for each condition (pain, touch, control). We utilized this approach (rather than single trial regressors) for subsequent analyses so as to obtain the most robust and consistent intra-participant patterns that could account for inter-participant variability. The z-stat maps were transformed to MNI standard space using the functional-to-standard transformation matrices produced by FNIRT during initial registration (see above) to create identically sized and shaped images (91 x 109 x 91 voxels), which were used for all subsequent analyses.

Second-level analyses were carried out to examine the interaction between individual levels of empathy measured using the Interpersonal Reactivity Index (IRI) and activation patterns from first-level z-stat maps. These results were thresholded with Randomise, using 30,000 permutations of group label. The resulting images were cluster corrected at a z-threshold of 2.3 and a p-value cutoff of 0.05.

### 2.3 MVPA analysis

#### 2.3.1 Somatomotor simulation analysis

All MVPA analyses were conducted using PyMVPA (Hanke et al., 2009), in combination with LibSVM’s (http://www.csie.ntu.edu.tw/~cjlin/libsvm/) implementation of the linear support vector machine (SVM), which allows for the integration of multiple binary classifiers using a “one-against-one method” (Hsu & Lin, 2002). For the SVM, we used a linear kernel and PyMVPA’s default regularization parameter C, which chooses this parameter automatically according to the Euclidean norm of the data. First, a whole-brain searchlight analysis (Kriegeskorte, 2006) was conducted to identify regions whose activation patterns permitted for a successful classification between the three somatosensory conditions (pain, touch, control). For this analysis, the input data to the classifier was a single 4D image file combining z-stat maps for each condition and participant, resulting in a total of 210 concatenated images (70 participants x 3 conditions). We centered an approximately spherical ROI (3 voxel radius) at each voxel in the brain and used the z-values from voxels within each sphere as a feature set in each of three binary support vector machine (SVM) classifications (pain vs. touch, pain vs. control, touch vs. control). Classification accuracy was assessed in a leave-one-participant-out cross-validation where the classifier was trained on all participants’ data except one, and then tasked with labeling the remaining participant’s data. Leave-one-out cross-validation was chosen since the MVPA analysis was not aimed at finding individual differences. The resulting average accuracy over all folds of the cross-validation was mapped to the center voxel of the sphere, resulting in a voxel wise map of across-participant classification accuracies.

#### 2.3.2 Somatomotor simulation: statistical thresholding

To enhance the replicability of our findings and isolate the most informative regions, we opted for a resel-wise Bonferroni correction in order to correct for multiple comparisons: we determined the total number of approx. 3 voxel radius non-overlapping spheres that could fit in the standard brain (~725) and divided 0.05 by this number to determine our alpha (6.89 *10-5)(Kaplan & Meyer, 2012). We established that this resel-wise Bonferroni corrected significance of 0.05 corresponded to a significant classification threshold of 46% (97 out of 210 total classifications) according to the binomial inverse cumulative distribution function.

#### 2.3.3 Dissimilarity searchlight analysis

A dissimilarity analysis was performed to investigate average dissimilarity in the brain’s representation of somatomotor states across various regions of interest. Within each participant, we used the uncorrected z-statistic for the three observed somatosensory conditions (Pain, Touch, Control). We used a modified representational similarity analysis technique that focused on calculating the difference between neural representations of our three somatosensory conditions. This analysis was performed across three contrasts: Pain vs. Control, Pain vs. Touch, and Touch vs. Control (seeFig. 1). Leveraging the same searchlight procedure (and parameters) outlined above, we calculated the Euclidean distance between vectorized voxel z-stat values within each sphere, resulting in three dissimilarity maps with values ranging between zero and two for each participant. The dissimilarity analysis was performed within each subject since it was not a classification analysis.

**Fig. 1.**
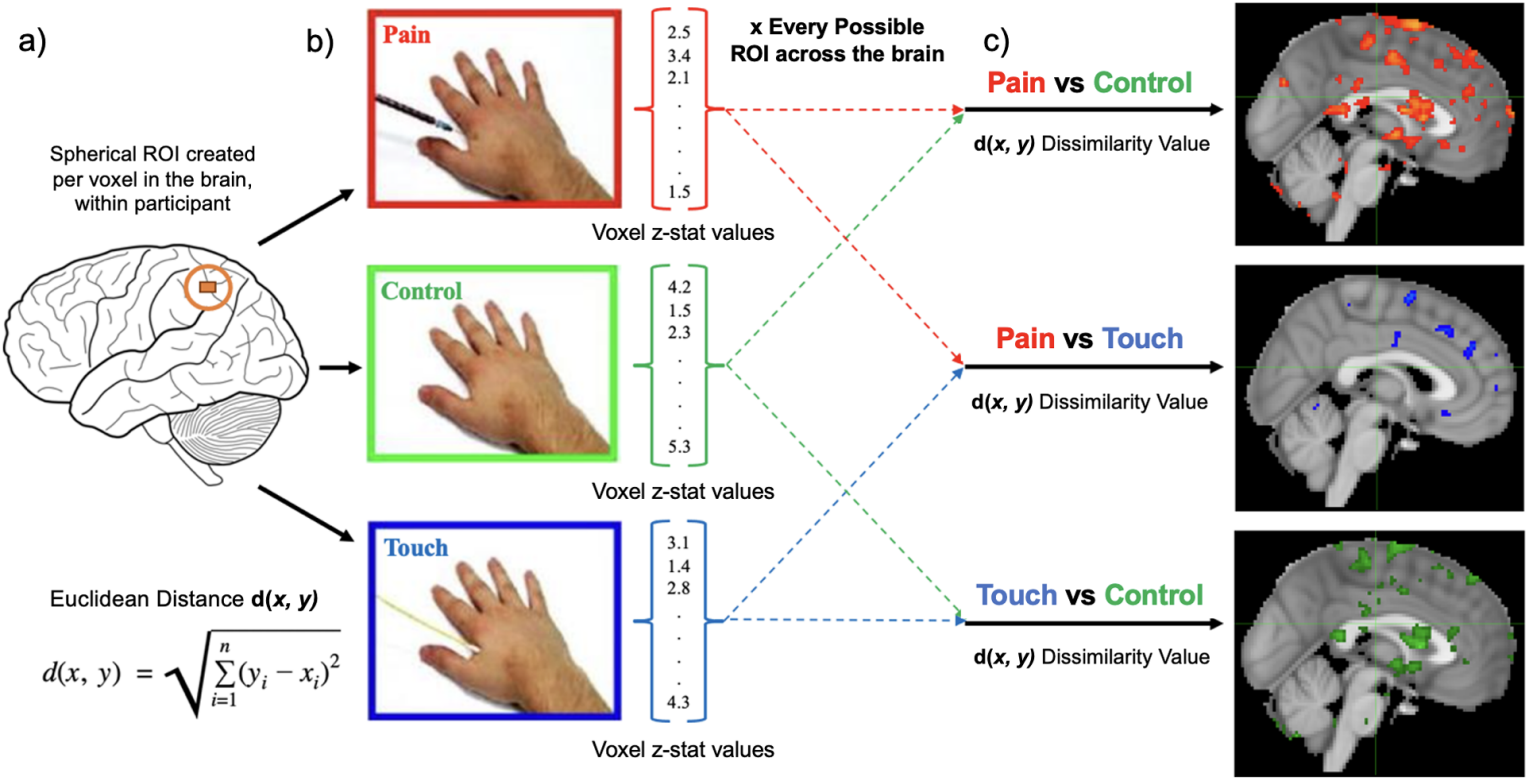
Dissimilarity searchlight analysis. (a) Within each participant, we created a spherical region of interest (ROI) around every voxel of the brain across the uncorrected z-statistic maps for all three SE conditions. Within these ROI’s, we vectorized the voxel z-stat values for the three conditions, (b) calculated the Euclidean distance between each pair of condition vectors (see equation above). This resulted in three dissimilarity values for each of the three contrasts, Pain vs. Control, Pain vs. Touch, and Touch vs. Control. Dissimilarity values were calculated for all possible ROIs in the brain and mapped to the center voxel of each; (c) this resulted in three dissimilarity maps per participant, averaged here to display regions in which patterns of neural activation showed higher levels of dissimilarity across the somatosensory states. We then regressed individual trait empathy scores from the Interpersonal Reactivity Index against these dissimilarity maps.

#### 2.3.4 Empathy correlation analysis

Each of the three dissimilarity searchlight outputs (Pain vs. Control, Pain vs. Touch, and Touch vs. Control) were correlated with each of the four IRI subscale scores across participants. This was performed in order to understand if the differences in activation across conditions had any relationship with subdimensions of empathy. Using FSL’s R*andomize*tool, we ran both positive and negative contrasts (correlations) for all four IRI subscales, demeaned, with all three dissimilarity contrasts across all participants. The analysis was performed with Threshold-Free Cluster Enhancement (TFCE), a thresholding method that encodes the level of cluster-like local spatial support in the voxel values rather than having to define an arbitrary cluster threshold, which has been shown to drastically affect results (S. M. Smith & Nichols, 2009). We reported the family-wise error corrected inverse p-value (1-p, where a value of 1 is the most significant).

## 3 Results

### 3.1 All results are reported within-text using the peak accuracy and coordinates for each region

#### 3.1.1 Univariate analyses

##### 3.1.1.1 Complementary univariate analyses were run for comparison

**Fig. 2.**
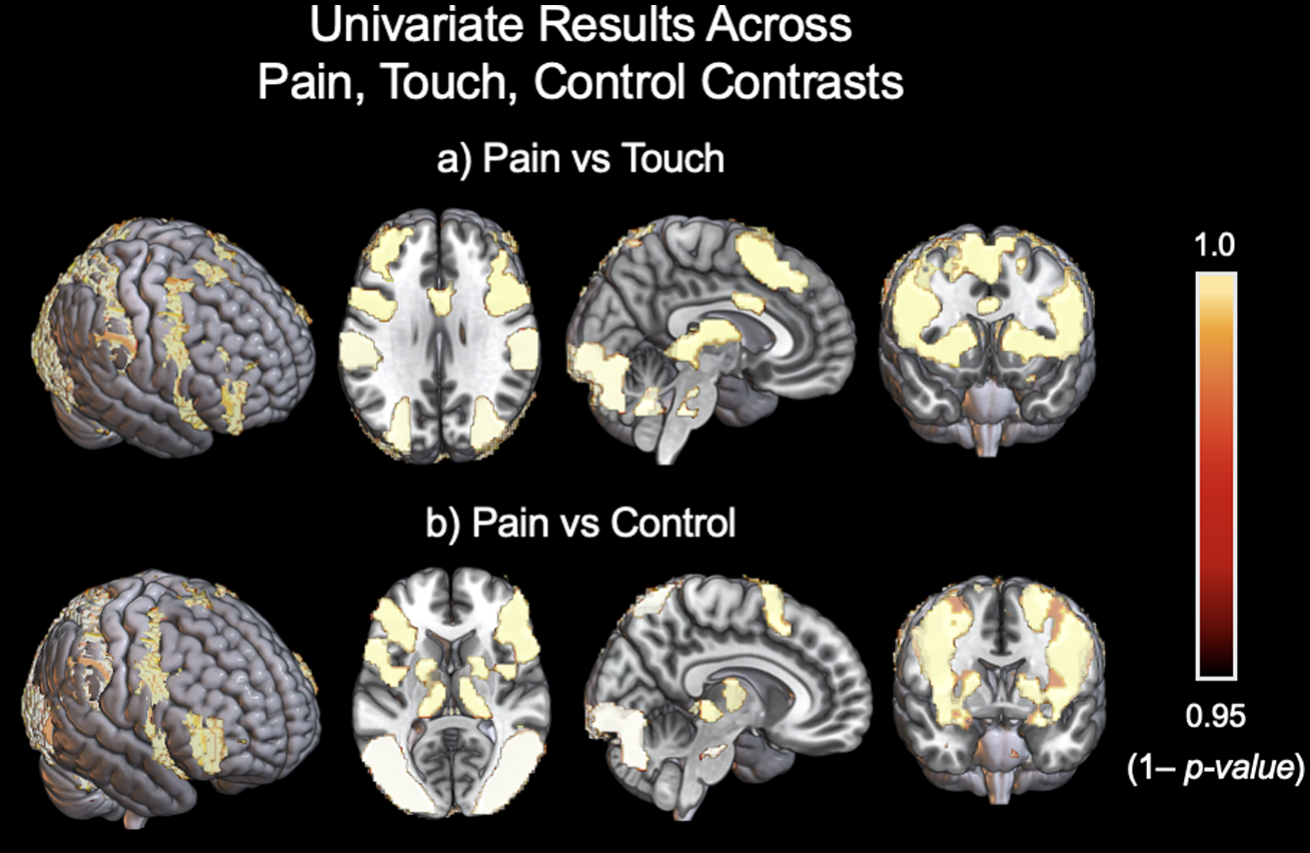
Univariate results for the pain condition contrasted with the touch condition and pain contrasted with control. The touch condition contrasted with control showed no significant group-level results. Relevant regions included the bilateral somatosensory cortex, bilateral insula, thalamus, and regions of the brainstem and cerebellum. The pain vs. touch contrast specifically displayed a significant cluster in the medial cingulate cortex.

Group mean results for pain, touch, and control conditions displayed activation across broad visual and somatosensory regions, as well as throughout multiple regions of the default mode network. None of these three conditions’ parameter estimate maps significantly correlated with any of the four IRI subscales. Group-level contrasts for pain vs. touch as well as pain vs. control showed similar regions of activation, with regions that activated more for the pain condition compared to touch, including somatosensory cortex as well as widespread areas of the visual cortex (Fig. 2). Areas that activated more for the touch condition compared to pain included the cerebellum and smaller regions of the somatosensory cortex. The pain vs. touch contrast positively correlated with the Fantasizing subscale of the IRI within areas of the precuneus (Z = 3.2, p = 0.05) and cingulate gyrus (Z = 2.6, p = 0.05) as well as a cluster in the cerebellum (Z = 2.9, p = 0.05). This contrast did not correlate positively or negatively with any of the other IRI subscales. The group-level contrast for pain vs. control mirrored the results of the pain vs. touch contrast, identifying similar but more widespread regions that activated more for the pain condition compared to the control condition. Areas that activated more for the control condition compared to the pain condition included a large portion of the precuneus as well as clusters throughout the somatosensory cortex. The touch vs. control condition did not show any significant activation as a group-level contrast. The pain vs. control and touch vs. control contrasts did not show significant correlations with any of the IRI subscales. We additionally contrasted (pain + touch) vs. control, which did not significantly correlate with any of the four IRI subscales.

#### 3.1.2 Whole brain searchlight

Multiple regions significantly supported classification of observed somatosensory states (i.e., Pain vs. Touch vs. Control), even using the more conservative (resel-wise Bonferroni-corrected) threshold (seeFig. 3).

**Fig. 3.**
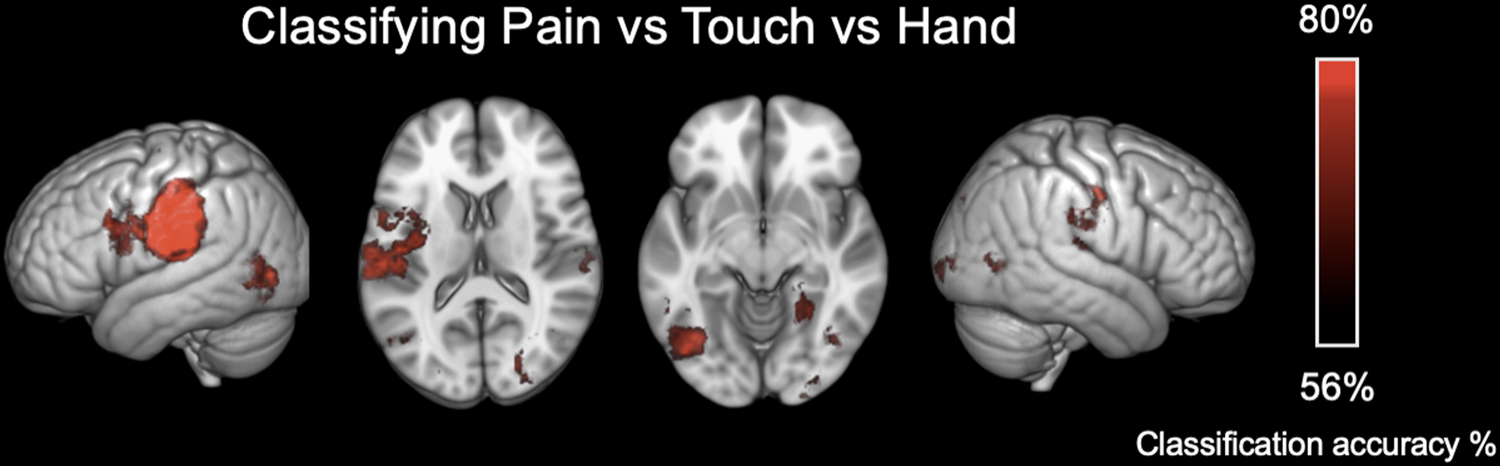
Accuracy of somatosensory classification searchlight. Areas in red indicate where patterns of activation best allow discrimination between hand-viewing conditions. These are strongest in inferior somatomotor, premotor areas contralateral to the observed hand, and bilateral visual areas.

These included Left Primary motor cortex (x = -44, y = -18, z = 42, accuracy = 66%), Primary Somatosensory Cortex (x = -44, y = -22, z = 36, accuracy = 66%), Secondary Somatosensory Cortex (x = -48, y = -22, z = 23, accuracy = 67%), Left Inferior parietal Lobe (x = -60, y = -30, z = 27, accuracy = 66%), Left Broca’s Ares BA44 (x = -49, y = 9, z = 27, accuracy = 62%), Left V5 (x = 40, y = -68, z = 0, accuracy = 61%), Right V5 (x = -38, y = -71, z = 3, accuracy = 61%), and Left SII/Insula (x = -37, y = 6, z = 16, accuracy = 61%).

### 3.2 Representational dissimilarity analysis

Regions that displayed the highest levels of dissimilarity varied across the three contrasts. Contrast maps displayed Euclidean values that ranged from 0 to 2 and were thresholded at 1.5 report areas with the highest levels of dissimilarity. However, overall dissimilarity for each contrast was seen in regions throughout the brain and overlapped with many of the areas that correlated with the IRI subscales (seeSupplemental Fig. 1&Table 1).

**Table 1.** Dissimilarity (Euclidean distance values).

Condition	Region of interest	Neurosynth associations	MNI coordinates	Value
Pain vs. Touch	Right Paracingulate Gyrus	Audiovisual perception, working memory	4 42 30	1.60
	Right Frontal Pole	Theory of mind, recognition	12 46 32	1.58
	Right Supplementary Motor Cortex	Motor planning & execution	4 2 62	1.52
Pain vs. Control	Left Frontal Pole	Accuracy, predictive judgments	-30 50 34	1.91
	Left Precentral Gyrus	Motor control, imitation	-60 0 36	1.88
Touch vs. Control	Left Frontal Pole	Information processing & retrieval	-34 46 38	1.85
	Left Precentral Gyrus	Premotor cortex, action representation	-60 -2 36	1.86

For the Pain vs. Touch contrast, this included the Paracingulate Gyrus (x = 4, y = 42, z = 30, ED = 1.60) as well as an area of the Supplementary Motor Cortex (x = 4, y = 2, z = 62, ED = 1.52). The Pain vs. Control and Touch vs. Control contrasts had highest levels of dissimilarity in similar areas of the Frontal Pole (Pain vs. Control: x = -30, y = 50, z = 34, ED = 1.91; Touch vs. Control: x = -34, y = 46, z = 38, ED = 1.85) and Precentral Gyrus (Pain vs. Control: x = -60, y = 0, z = 36, ED = 1.88; Touch vs. Control: x = -60, y = 2, z = 36, ED = 1.86).

The Pain vs. Control contrast showed significant positive correlation with the Empathic Concern subscale, most prominently in the bilateral Postcentral Gyrus (Left: x = -56, y = -20, z = 38, FWE-corrected p < 0.01; Right: x = 50, y = -26, z = 56, FWE-corrected p < 0.03). There was also significant correlation in areas of the Superior Temporal Gyrus (x = 48, y = -2, z = 12, FWE-corrected p < 0.03), Frontal Pole (x = 26, y = 48, z = 32, FWE-corrected p < 0.5), and Paracingulate Gyrus (x = 2, y = 46, z = 12, FWE-corrected p < 0.5) (seeFig. 4a). The Touch vs. Control condition showed positive correlations with multiple IRI subscales, namely Empathic Concern, Fantasy, and Personal Distress (seeFig. 4b). Touch vs. Control was most significantly correlated with the Fantasy subscale, specifically in the right Lateral Occipital Cortex (x = 24, y = -78, z = 22; FWE-corrected p < 0.02). It showed significant correlation with the Empathic Concern in the left Lateral Occipital Cortex (x = -30, y = -74, z = 38; FWE-corrected p < 0.05) and with the Personal Distress subscale in the Frontal Pole (x = 20, y = 40, z = -16; FWE-corrected p < 0.05). The Pain vs. Touch contrast did not show any significant correlations with either of the four IRI subscales across participants. None of the negative correlations were significant across any contrast and empathy subscale (seeTable 2).

**Fig. 4.**
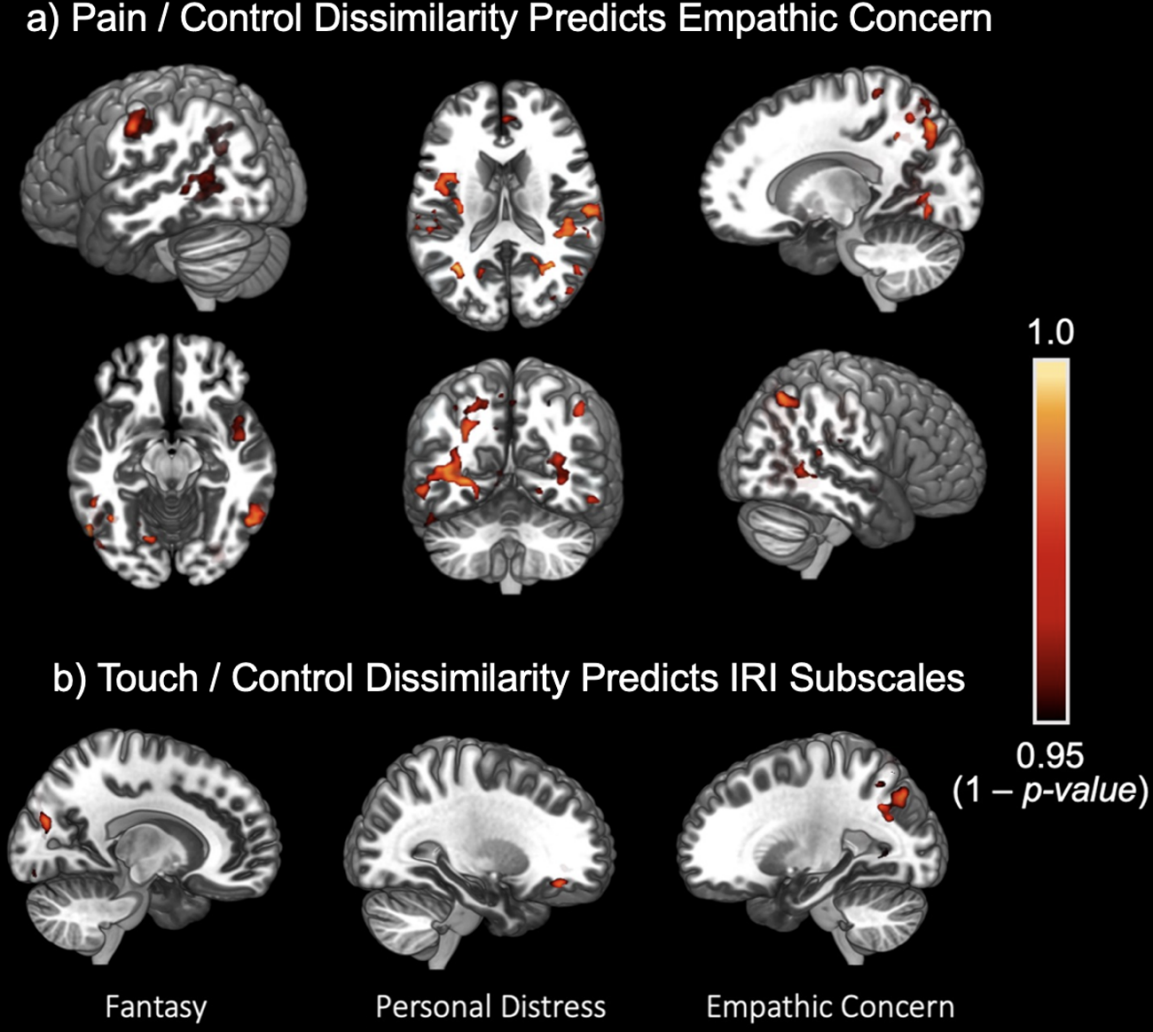
Areas where higher levels of dissimilarity in activation patterns were positively correlated with empathic subdimensions. a) Pain vs Control contrast correlated with the Empathic Concern subscale. b) Touch vs Control contrast correlated with the Fantasizing, Personal Distress, and Empathic Concern subscales.

**Table 2.** Empathy trait correlations.

Condition	IRI subscale	Region of interest	Neurosynth associations	MNI coordinates	p-value
Pain vs. Control	Empathic Concern	Left Postcentral Gyrus	Primary somatosensory cortex	-56 -20 38	0.01
		Right Postcentral Gyrus	Somatosensory cortex (hand area)	50 -26 56	0.03
		Superior Temporal Gyrus	Language & music perception	48 -2 -12	0.03
		Right Frontal Pole	Response inhibition	26 48 32	0.04
		Right Paracingulate Gyrus	Decision-making, conflict	2 46 12	0.04
Touch vs. Control	Fantasy Scale	Right Lateral Occipital Cortex	Spatial & motion processing	24 -78 22	0.02
Touch vs. Control	Personal Distress	Orbital Frontal Cortex	Decision-making & value	20 40 -16	0.02
Touch vs. Control	Empathic Concern	Left Lateral Occipital Cortex	Memory retrieval, simulation	-30 -74 38	0.02

## 4 Discussion

We found multiple regions in which pattern dissimilarity of BOLD signal changes across voxels while observing distinct somatosensory experiences allowed us to classify the states being observed and predict individual differences in empathic traits. Importantly, the multivariate analyses conducted improved upon our initial univariate findings by identifying regions in which the granularity of neural representations related to individual empathic differences. Broadly, the fact that the most informative regions were in the participants’ own somatosensory and interoceptive cortices suggests that some form of simulation is at play during these experiences. Specifically, the locations of regions implicated in predicting empathic function may be informative, as we will discuss below.

### 4.1 Univariate analyses

Regions that were active for the pain condition compared to both touch or control conditions were widespread throughout the brain and provided little specificity in terms of how these regions may relate to individual empathic traits. These regions spanned visual and somatosensory cortices, as would be expected when viewing stimuli that varied in terms of the sensory experience. Few of these results provided any insight into individual empathic differences, with only the Pain vs. Touch contrast significantly correlating with any of the four IRI subscales in specific nodes of the default mode network. Importantly, when correlating both Pain and Touch vs. Control with all four subscales, we did not have any significant results, as well as with the Touch vs. Control contrast. However, the lack of significant correlations and conflicting results suggested the need for a more granular investigation into the underlying patterns of these neural representations.

### 4.2 Classification of vicarious states

Observed somatosensory states occurring in response to a right hand were best classified by patterns in contralateral primary and secondary somatosensory cortex,*the same areas that would be active were the participant experiencing the sensory stimuli themselves*. This supports a simulationist interpretation of this finding (Oliver et al., 2018;Vollberg et al., 2021). Many previous studies have found that there is significant overlap between networks supporting pain, and empathy upon observing the same type of pain (Lamm et al., 2011). In addition, classification was informed by areas traditionally associated with the human mirror neuron system (inferior parietal lobe and broca’s area BA44). These results agree with prior findings showing that the human mirror neuron system supports observation of others’ internal states, and that observed somatomotor experience affects motor cortex excitability (Avenanti et al., 2005;Fadiga et al., 1995).

### 4.3 Dissimilarity analysis

Across the three dissimilarity contrasts, the Pain vs. Control contrast was most significantly correlated with empathic concern, specifically based on patterns of activity in the left Postcentral Gyrus. These results indicate that larger differences in patterns of activity within sensory regions, such as the primary somatomotor cortex, show a relationship with higher self-reported measures of “other-related” sympathetic feelings. This further highlights the influence of bottom-up sensory representations of somatosensory states. The Touch vs. Control contrast was correlated with both empathic concern as well as the fantasy subscale, however in higher level visual areas, primarily the Lateral Occipital Cortex. This emphasizes a separate sensory input that may be affecting individual empathetic traits. Interestingly, no region’s degree of dissimilarity was negatively associated a sub-facet of empathy, suggesting that the uniqueness of patterns generated for observed pain, touch, and body part viewing may generally be associated with increased tendencies across subfacets of empathy.

We observe correlations with areas of Pain vs. Control dissimilarity, and Touch vs. Control dissimilarity, but not Pain vs. Touch, supporting distinct hierarchies for tactile and nociception, additionally suggesting that fine-grained vicarious nociception is less relevant for aspects of empathy other than empathic concern. These results underline the computational advantage of using novel multivariate methods as they elucidate findings that were unable to be identified with standard univariate methods. With univariate analyses, we did not find any significant results with the Touch vs. Control contrast, either on a group level or across correlations with IRI subscales. Using our dissimilarity analysis, we found group-level differences in Touch vs. Control as well as significant correlations with IRI subscales of empathic concern and fantasy, presumably due to these results relying upon the grain of neural patterns rather than general levels of activation.

Global theories of brain organization converge on the idea that the brain represents aspects of the world via nested, hierarchical imagetic patterns. These represent, in ascending order, increasingly abstract, diverse, and complex features of the world ranging from basic sensory features all the way up to cognitive models of other living agents. This scheme relies not only on abstraction but also on integration—thus, areas can be understood not only by their level of abstraction/complexity, but also by the degree of information convergence. These may include integrated affective interoceptive inferential cognitive information that is quite complex. Hence, examining which systems’ representations are most associated with different types of empathic ability may shed light on the modalities and level of abstraction that are employed in each sub process.

Thus, it is interesting to note that fantasizing seems to rely on the distinctiveness of representations in areas involved in visual perception such as the lateral occipital cortex, personal distress on distinctiveness in affective areas (VMPFC). Conversely, we can see how somatomotor and visceral representations seem to be useful for empathic concern coupled with representations and areas involved in the high-level representation of affective states. Thus, the hierarchical position, the level of integration represented putatively by implicated areas, and complexity of these patterns are informative for understanding how a given process is served by the brain. While fMRI studies typically do not show such lateralization in “mirroring” responses, such patterns have been observed in TMS studies (Avenanti et al., 2005,^2006^). Our dissimilarity-based method may detect lateralization (and actual sensorimotor simulation processes) better than univariate methods, suggesting intriguing conclusions about neural representations of others’ states within neuroimaging data. However, further study (using opposite-side hand images) will be necessary to demonstrate this conclusively.

Limitations of this study include the nature in which empathy measures were collected. Importantly, we collected only trait empathy measures reliant upon self-report surveys and had no measures of state empathy. Additional studies could expand upon our findings by including state-level empathy measures along with the self-report IRI scale, such as having participants report their empathic state at various points within the scan. This could take multiple forms, such as having participants describe their emotional state in the moment, the amount of pain they perceive in others, or the extent they are feeling distressed by the vicarious experience. Our use of a block design limits our interpretation of parameter estimates arising from each block, which can be due to overall differences throughout or changes throughout the block. We acknowledge the possibility that individual differences in empathic ability may be observable in these within-block dynamics but have elected not to examine this in the current study.

Overall, these results demonstrate that the degree of self-reported empathic tendencies is strongly correlated with the distinctiveness of neural somatic representations. Follow-up work could examine neural dissimilarity between an individual observing pain and them actually receiving painful simulation to a) account for individual differences in empathic traits and b) track longitudinal changes in cognitive vs. affective empathy training. In conclusion, our findings not only illustrate the importance of leveraging advanced multivariate techniques to understand complex aspects of cognition, but also provide evidence for simulationist theories of empathy. This is augmented by the additional finding that distinctiveness of neural patterns within specific sets of regions relates to individual differences in trait empathy, further suggesting that effective empathy relies on the fidelity of multiple facets of individuals’ internal simulation of observed states.

The approach described here provides useful information about neuropsychological correlates complementary to univariate analyses of activation. This approach may be fruitfully employed to assess outcomes, ability, and dysfunction as well as generally elucidate brain behavior relationships involved in complex processes. These findings provide a framework for understanding conditions of social cognition as arising from impaired capability for experience (as seen in psychopathy or autism). Representational dissimilarity may present a rare case of an analytical approach to complex neuroimaging data that reflects a computational property of the underlying substrate.

## Supplementary Material

Supplementary Material

## Data Availability

All data and code are hosted on the Open Science Foundation, identifier: DOI 10.17605/OSF.IO/DAQ9Z.
