# Supplementary material for: Empathy from dissimilarity: Multivariate pattern analysis of neural activity during observation of somatosensory experience
Source: Imaging Neurosci (Camb). 2024 Mar 19;2:imag-2-00110. doi: 10.1162/imag_a_00110 (PMC12247545; doi:10.1162/imag_a_00110)
Supplement: Supplementary Material [file imag_a_00110-supp.pdf]

Supplemental Materials

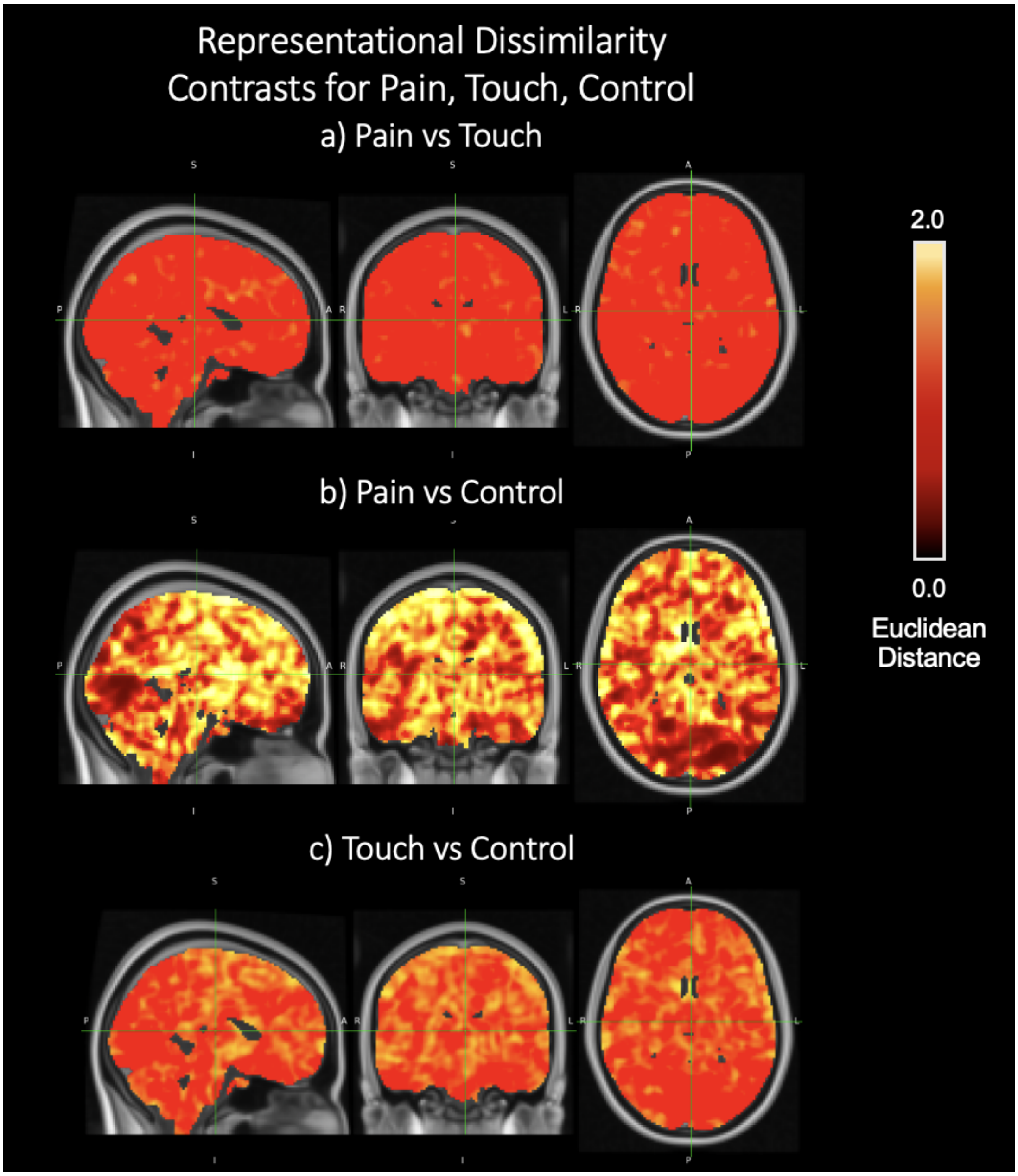

Supplemental Figure: Dissimilarity results for each contrast (Pain vs Touch, Pain vs Control, Touch vs Control), displaying the Euclidean distance maps. Euclidean distance was measured within each searchlight sphere across the brain, calculating the mathematical distance between the activation values within that sphere for one condition and another condition, per participant. These maps were aggregated across participants to create group-level maps. Here, we display the entire range of Euclidean distance values (0 to 2), where the yellow/white regions display areas with the highest level of dissimilarity and red/darker regions display regions with lower levels of dissimilarity.
